# The Role of Phenolic Profile of Salt-Stressed Duckweed (*Lemna minor*) in Synthesis and Biological Activity of Green ZnO Nanoparticles

**DOI:** 10.3390/molecules31132326

**Published:** 2026-07-02

**Authors:** Nikola Stamenković, Filip Nikolić, Aleksandar Matić, Dragana Antonić Reljin, Marija Milovančević, Danijela Paunović, Olga Radulović

**Affiliations:** Institute for Biological Research “Siniša Stanković”—National Institute of the Republic of Serbia, University of Belgrade, 11060 Belgrade, Serbia; nikola.stamenkovic@ibiss.bg.ac.rs (N.S.); filip.nikolic@ibiss.bg.ac.rs (F.N.); aleksandar.matic@ibiss.bg.ac.rs (A.M.); dragana.antonic@ibiss.bg.ac.rs (D.A.R.); marija.djuric@ibiss.bg.ac.rs (M.M.); danijela.paunovic@ibiss.bg.ac.rs (D.P.)

**Keywords:** duckweed, nanoparticles, phenolic compounds, green synthesis, antimicrobial activity

## Abstract

This study investigated whether salinity during cultivation of the aquatic plant *Lemna minor* (duckweed) influences the phytochemical composition of plant extracts and the properties of green-synthesized zinc oxide nanoparticles (ZnO NPs). Duckweed was cultivated under 0, 10, and 100 mM NaCl, followed by Orbitrap metabolomic profiling, nanoparticle synthesis, physicochemical characterization, and evaluation of antioxidant and antimicrobial activities. Orbitrap analysis revealed pronounced salinity-dependent changes in extract composition, including increased abundance of several flavonoids, glycosylated flavones, and hydroxycinnamic acid derivatives in the order 0 < 10 < 100 mM. ZnO nanoparticle formation was supported by UV–Vis spectroscopy, which showed characteristic absorption features around 360 nm, and by powder X-ray diffraction (PXRD), which indicated the predominance of the hexagonal wurtzite ZnO phase in all samples. SEM–EDS analysis revealed Zn- and O-rich materials consisting of micron-scale aggregates and finer submicron structures. Raman spectra were dominated by fluorescence, which increased with salinity treatment and may reflect differences in surface-associated phytochemicals rather than substantial changes in the ZnO crystal structure. Nanoparticles synthesized using extracts from salt-stressed duckweed exhibited higher total phenolic content (up to 66.79 ± 0.15 µM GAE g^−1^), antioxidant activity (up to 55.01 ± 0.21%), and antimicrobial activity against *Staphylococcus haemolyticus* D4-2-100/1 (inhibition zone up to 1.55 ± 0.05 cm). Although the mechanisms underlying these differences remain to be fully elucidated, the results suggest that salinity-induced changes in duckweed metabolism may influence the biological properties of the resulting nanomaterials. Overall, this study highlights the potential of manipulating cultivation conditions to modulate plant extract composition and, consequently, influence the characteristics and functionality of green-synthesized ZnO nanoparticles.

## 1. Introduction

Green synthesis of nanoparticles using plant extracts has emerged in the last decade as a potentially sustainable, cost-effective, and biologically justifiable alternative to conventional chemical and physical methods [1,2]. This technology relies on the interaction of plants’ secondary metabolites with metal ions as stabilizing and reducing agents, which enable the formation of nucleation centers and thus initiate the generation of nanoparticles [3,4]. During this reaction, stabilizing (or capping) agents further adsorb to the nanoparticle surface and prevent uncontrolled aggregation by providing electrostatic repulsion or steric hindrance, ensuring colloidal stability and controlling particle size and morphology [1,2,3,5,6]. This biologically mediated process not only offers a comparatively greener alternative to conventional methods but also produces nanoparticles with surface-bound biologically active phytochemicals, which can enhance the nanoparticles’ biocompatibility and antimicrobial activity [5,6,7]. The efficiency and characteristics of this synthesis are directly influenced by the phytochemical composition of the extract, which, in turn, is shaped by the plant’s physiological state and environmental conditions. In this context, *Lemna minor* L. (duckweed) is gaining recognition as a biofactory for green nanoparticle synthesis due to its rapid vegetative propagation, minimal cultivation requirements, and dynamic phytochemical profile [1,8,9].

The metabolic plasticity of duckweed is well known, especially in responses to salt stress and starvation [10,11,12]. However, its role in the biosynthesis of duckweed-derived zinc oxide nanoparticles (ZnO NPs) is underexplored [1,8,9]. While multiple studies of green nanomaterial synthesis have focused on optimizing reaction parameters, nanoparticle morphology, and bioactivity, a critical variable, such as the physiological and environmental conditions under which plants are cultivated, remains largely underexplored [1,12,13]. Plant growth conditions are known to influence the concentration and composition of secondary metabolites, which in turn directly affect nanoparticle synthesis efficacy, stability, and functionality [9,14,15]. The effects of microbes are also overlooked in green synthesis studies, where biomass is harvested from decidedly non-sterile conditions [1,8,16]. However, the microbiome of plants is well known to modulate phytochemistry [17,18]. Moreover, non-sterile conditions imply other unexpected factors, e.g., chemical impurities, which may hinder the synthesis. Therefore, in this study, the duckweed biomass was deliberately grown in vitro. Addressing these gaps is important for developing standardized and predictable green synthesis protocols, as the phytochemical profile of the extract is not static but dynamically modulated by environmental cues.

Although silver is the most common metal for green nanomaterial synthesis [2,19,20,21], ZnO NPs offer several potential advantages [5,22,23,24,25]. While silver nanoparticles (AgNPs) are known for potent antimicrobial effects, their cytotoxicity, environmental persistence, and higher cost limit their applicability [2,19,20,26]. In contrast, ZnO is classified as “Generally Recognized as Safe” (GRAS) by the U.S. FDA, enabling its use in food, biomedical, and environmental systems without the same regulatory concerns [27]. ZnO NPs exert broad-spectrum antimicrobial activity via Zn^2+^ ion release, reactive oxygen species (ROS) generation, and membrane disruption; all achieved with lower ecotoxicological risks than silver [28,29,30]. Unlike silver, zinc is also an essential micronutrient, enhancing its biological compatibility and reducing off-target toxicity [27]. Moreover, the biosynthesis of ZnO NPs is more compatible with mild, aqueous extraction systems. Zn^2+^ ions chelate efficiently with polyphenols, flavonoids, and carboxylic acids, facilitating controlled crystallization and surface capping under ambient conditions [1,9]. ZnO also demonstrates superior photocatalytic efficiency, enabling multifunctional use in UV protection, water disinfection, and biofilm disruption [22,23,24,31]. Together, these properties make ZnO NPs a sustainable and functionally superior alternative to AgNPs, especially when synthesized from plant biomass under defined physiological conditions [9].

Finally, the rise of multidrug resistance and hospital-acquired infections highlights the need for novel antimicrobial agents [3]. The increasing prevalence of multidrug-resistant (MDR) pathogens poses an urgent threat to global public health, with estimates suggesting that antimicrobial resistance (AMR) could cause up to 10 million deaths annually by 2050 if left unaddressed [32]. Green nanoparticles possibly offer such a solution, as a standalone or supplementary agent with antimicrobial activity.

In this observational and exploratory study, we investigated how NaCl-induced stress (0, 10, and 100 mM) affects the metabolomic composition of duckweed extracts and modulates the synthesis of ZnO NPs. We tested this green synthesis strategy in small-scale, in vitro conditions to test its basic feasibility. To this end, high-resolution Orbitrap mass spectrometry was employed to quantify and identify key stress-responsive phenolic compounds. The optical properties of nanoparticles were monitored via UV–Vis spectroscopy, while X-ray diffraction (XRD) was employed to assess nanoparticle crystallinity and phase purity.

The antimicrobial activity of synthesized ZnO NPs was evaluated using a well diffusion method against two opportunistic bacterial pathogens with potential to acquire MDR: *Staphylococcus haemolyticus* and *Serratia fonticola*. This research integrates plant stress physiology, metabolomic profiling, green chemistry, crystallography, and microbiology to develop an eco-sustainable nanoparticle synthesis approach based on duckweed cultivation. It supports a broader vision of optimizing nanomaterial fabrication through biochemical modulation and biomass valorization and bringing the process closer to the ideals of the circular economy. Ultimately, this study provides insight into the use of aquatic plants as renewable sources of bioactive nanomaterials with antimicrobial potential.

## 2. Results

### 2.1. Phytochemical Analysis of the Duckweed Extract

Targeted Orbitrap analysis was employed primarily to characterize the chemical profiles of duckweed extracts exposed to salt stress. In total, 30 naturally occurring compounds were identified by HPLC–Orbitrap-Ms analysis (Table 1). The majority of identified compounds had an aromatic structure, with the exception of jasmonic and geniposidic acid.

**Figure 1:**
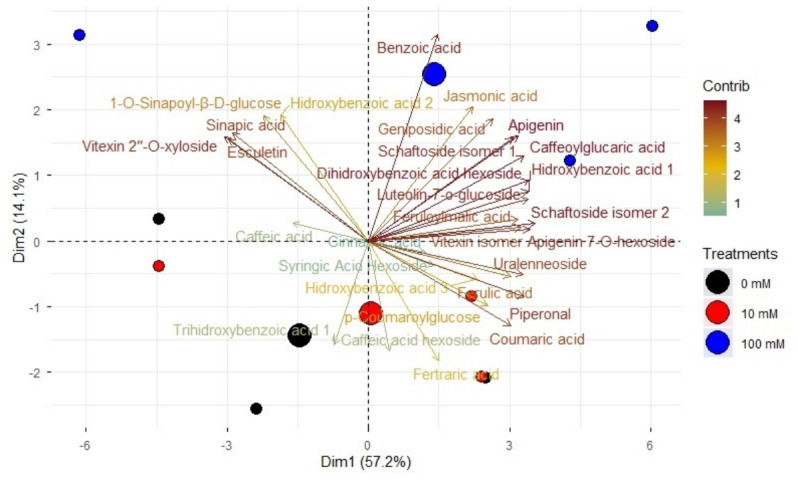
Biplot representing principal component analysis (PCA) plot of 30 compounds isolated from duckweeds exposed to salt stress, combined with duckweed response distribution to salinity stress. The data distribution and vector plots were constructed in R, using FactoExtra and FactoMineR 2.15 packages.

Upon exposure to increasing concentrations of NaCl (0, 10, and 100 mM), the duckweed extracts exhibited significant changes in their specialized metabolite profiles (Figure 1).

The analysis for all treatments shows that the two principal components (PC1 and PC2) explain 71.3% of total data variability, with PC1 explaining 57.2% and PC2 explaining 14.1% (Figure 1). PC1 was mainly influenced by schaftoside isomers 1 and 2, apigenin and apigenin 7-O-hexoside, hydrohydroxybenzoic acid 1, dihydroxybenzoic acid hexoside, luteolin-7-O-glucoside, piperonal, caffeoylglucaric acid, uralenneoside, feruloylmalic acid, vitexin and vitexin 2″-O-xyloside, coumaric acid, ferulic acid and esculetin. The component PC2 was significantly correlated with benzoic acid, jasmonic acid, hydroxybenzoic acid 2, sinapic acid, 1-O-sinapoyl-β-D-glucose, geniposidic acid, caffeic acid hexoside, fertraric acid, apigenin, vitexin 2″-O-xyloside, trihydroxybenzoic acid 1, schaftoside isomer 1, and esculetin (Supplementary Figure S1A). Data from duckweeds treated with 0 and 10 mM NaCl were mainly grouped between the center and the negative end of the y-axis. The only difference between these two groups is that data from duckweeds treated with 0 mM were pointed toward negative values of the x-axis, while data from duckweeds treated with 10 mM gravitated toward positive values of the x-axis. Based on the PCA of phenolic profiles of duckweeds, it can be concluded that duckweeds grown at 10 mM NaCl have a similar phenolic profile to duckweeds grown in control conditions due to overlapping. Duckweeds treated with 100 mM NaCl formed a distinctive group located toward the positive end of the y-axis and exhibited a different phenolic profile (Figure 1). Based on the directions of vectors representing phenolic compounds, it can be concluded that benzoic acid, jasmonic acid, hydrohydroxybenzoic acids 1 and 2, geniposidic acid, vitexin 2″-O-xyloside, sinapic acid, 1-O-sinapoyl-β-D-glucose, esculetin, apigenin, schaftoside isomer 1, and caffeoylglucaric acid are associated with salt stress response. On the other hand, piperonal, coumaric acid, fertratic acid, caffeic acid hexoside, trihydroxybenzoic acid 1, ferulic acid, hydrohydroxybenzoic acid 3, syringic acid hexoside, and caffeic acid are correlated with stress-free (0 mM) and low-intensity stress (10 mM) conditions.

**Figure 2:**
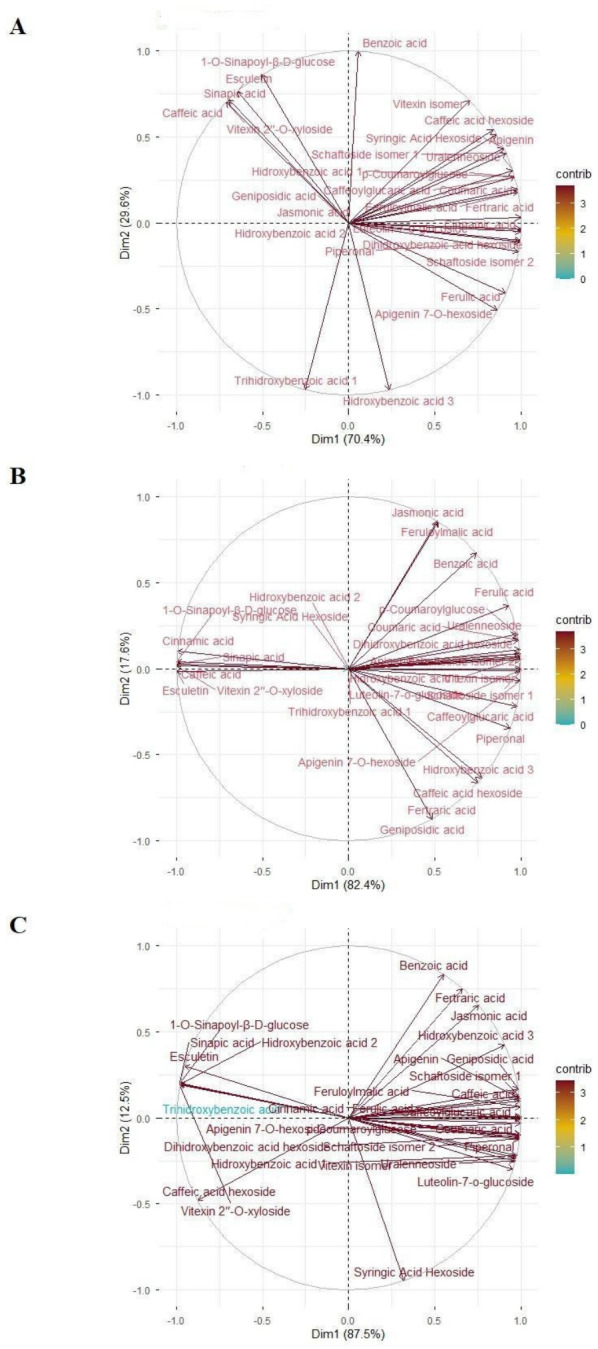
Vector plots of variables corresponding to different salt treatments: (**A**)—0 mM; (**B**)—10 mM; (**C**)—100 mM NaCl. Plots were constructed in R using Factoextra and FactoMineR packages.

The PCA for each treatment further revealed the separation of 0, 10, and 100 mM groups and highlighted the increase in the relative contribution of PC1 to total data variability from 70.4% at 0 mM (Figure 2A) to 82.4% at 10 mM (Figure 2B) and 87.5% at 100 mM (Figure 2C).

For the 0 mM group, the PC1 was determined by ferulic acid, cinnamic acid, luteolin-7-O-glucoside, hydrodihydroxybenzoic acid hexoside, piperonal, schaftoside isomers 1 and 2, feruloylmalic acid, coumaric acid, hydroxybenzoic acid 1, p-coumaroylglucose, caffeoylglucaric acid, uralenneoside, syringic acid hexoside, apigenin and apigenin 7-O-hexoside (Figure 2A). Meanwhile, PC2 was significantly correlated with benzoic acid, hydroxybenzoic acid 3, trihydroxybenzoic acid 1, sinapic acid and 1-O-sinapoyl-β-D-glucose, esculetin, vitexin isomer and vitexin 2″-O-xyloside, and caffeic acid (Supplementary Figure S1B).

For duckweeds grown at 10 mM NaCl, the PC1 and PC2 accounted for 82.4% and 17.6% of the total data variability (Figure 2B). Luteolin-7-O-glucoside, schaftoside isomers 1 and 2, esculetin, caffeic acid and its hexoside, vitexin isomer and its 2″-O-xyloside, sinapic acid and its 1-O-β-D-glucoside, hydrohydroxybenzoic acid 1, apigenin and its 7-O-hexoside, cinnamic acid, dihydroxybenzoic acid hexoside, uralenneoside, coumaric acid and p-coumaroylglucose influenced the PC1. Geniposidic acid, fertraric acid, jasmonic acid, feruloylmalic acid, benzoic acid, caffeic acid hexoside, and hydroxybenzoic acid 3 determined the PC2 (Supplementary Figure S1C).

**Figure 3:**
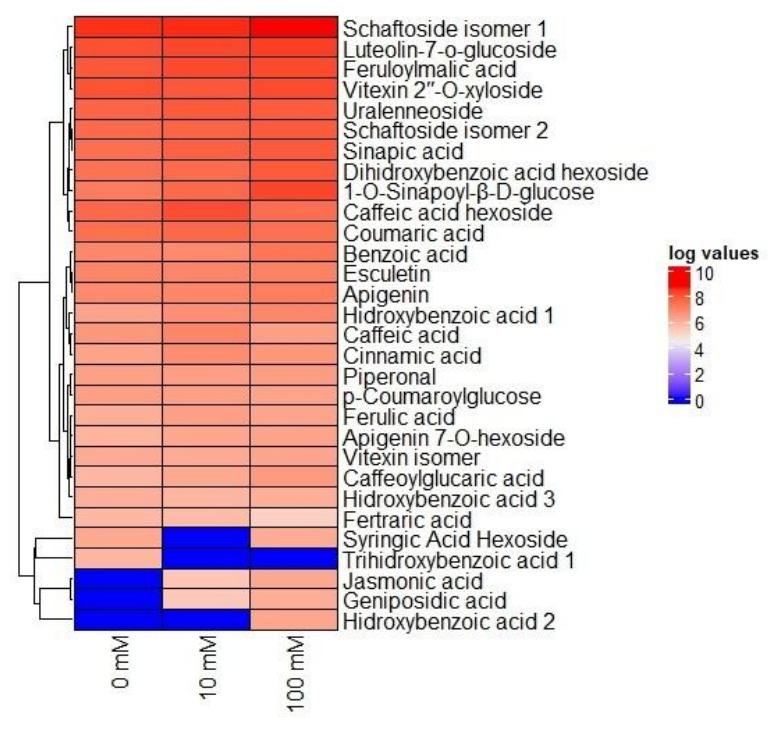
Cluster heatmap analysis representing the changes in 30 compounds isolated from duckweed. Clustering is described by the dendrogram on the left. Red color indicates the increase in different parameters, and blue color indicates the decrease. The heatmap was constructed in R using complexheatmap package.

For duckweeds grown at 100 mM NaCl, PC1 and PC2 accounted for 87.5% and 12.5% of the total data variability (Figure 3). The component PC1 was mainly shaped by ferulic acid, cinnamic acid, caffeoylglucaric acid, coumaric acid and p-coumaroylglucose, apigenin and apigenin 7-O-hexoside, caffeic acid, dihydroxybenzoic acid hexoside, schaftoside isomers 1 and 2, piperonal, geniposidic acid, vitexin isomer and vitexin-2″-O-xyloside, hydroxybenzoic acid 2, sinapic acid and 1-O-sinapoyl-β-D-glucose, hydroxybenzoic acid 1, uralenneoside and luteolin-7-O-glucoside. On the other hand, PC2 was significantly determined by syringic acid hexoside, benzoic acid, fertraric acid, jasmonic acid, and caffeic acid hexoside (Supplementary Figure S1D).

For heatmap analysis, 30 compounds were used, of which 28 were phenolics (Figure 3). Based on the change in log_10_ values, parameters can be classified into two clusters. Cluster I includes 5 phenolic compounds, which were missing in some treatments. The remaining 25 compounds, which are found in all treatments, form cluster II. Cluster II can be further divided into subclusters II_a_ and II_b_. Subcluster II_a_ encompasses compounds whose content was not significantly influenced by treatments. Accordingly, phenolic compounds whose content was generally increasing with implemented concentrations of NaCl during treatments were grouped in subcluster II_b_. In this group, schaftoside isomers 1 and 2, luteolin-7-O-glucoside, feruloylmalic acid, vitexin 2″-O-xyloside, uralenneoside, sinapic acid, dihydroxybenzoic acid hexoside, 1-O-sinapoyl-β-D-glucose, caffeic acid hexoside, and coumaric acid were identified.

### 2.2. Spectroscopic UV–Vis Characterization of ZnO Green Nanoparticles Synthesized from the Duckweed Extract

**Figure 4:**
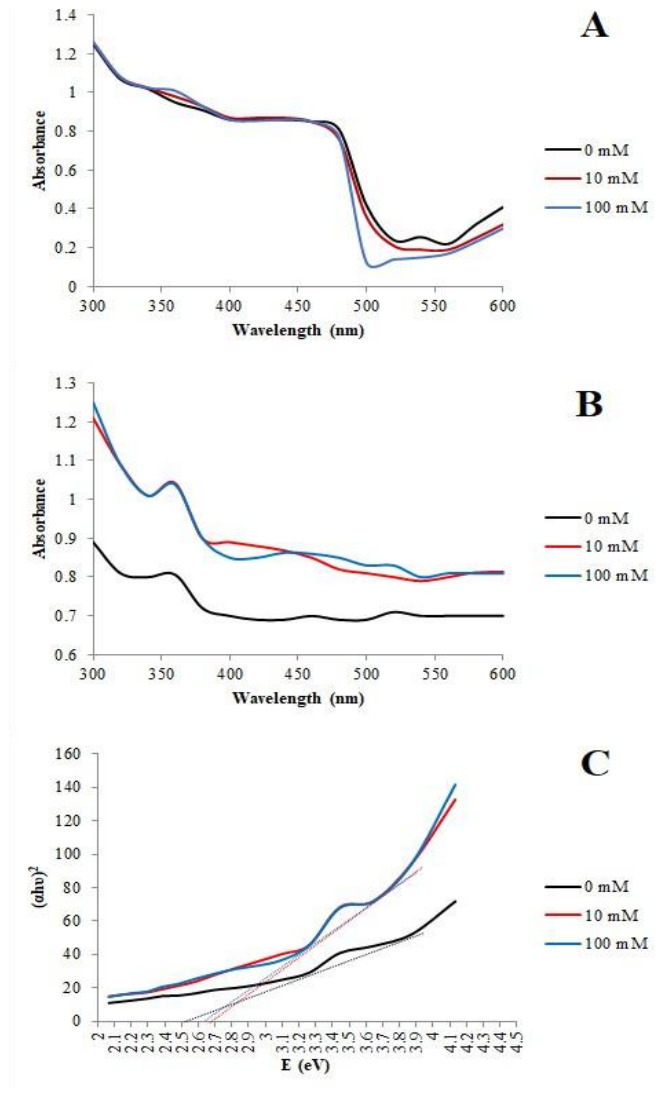
UV–Vis spectra of the duckweed extract (**A**) and ZnO-NP solution (**B**) with the corresponding Tauc plot (**C**).

To assess the optical properties of the solution with ZnO green nanoparticles synthesized from the duckweed extract, and to compare them with the optical properties of the mentioned extract, UV–Vis spectroscopy was performed (Figure 4).

The UV–Vis absorption spectra of the ethanolic extract of duckweed at varying concentrations of the salt treatment (0 mM, 10 mM, and 100 mM) are shown in Figure 4A. All spectra exhibited an absorption maximum in the approximate range of 320–380 nm and a spectral valley with a minimum around 520 nm.

The UV–Vis spectra of the analyzed duckweed-derived nanomaterial had characteristic absorbance peaks (λ*_max_*) shifted toward the visible part of the spectrum, most notably around 360 nm (Figure 4B). The absorbance maxima also increased with the salinity increment from 0 to 10 and 100 mM NaCl. The spectral valley around 520 nm was absent.

The Tauc plot deduced from the UV–Vis spectra of ZnO-NPs revealed that the estimated band gap energy (*E*_g_) increased with increasing exposure to salt stress during duckweed cultivation, from approximately 2.5 eV for 0 mM to 2.65 eV for 10 and 100 mM samples (Figure 4C).

### 2.3. Analysis of ZnO NPs with X-Ray Diffraction on Powdered Material (PXRD)

**Figure 5:**
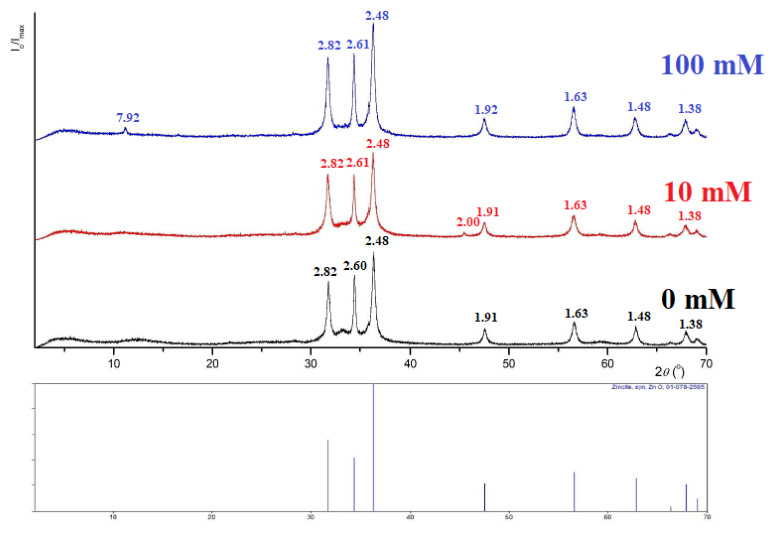
Comparison of diffractograms of three nanomaterial samples synthesized from the extract of duckweed exposed to 0, 10, or 100 mM NaCl, with standard ZnO peaks (diffractogram at the bottom). The d-values (in Å) are shown above each peak.

To test the presence of crystalline material in synthesized samples, a PXRD was performed. In all three tested samples, hexagonal ZnO crystals were detected (Figure 5).

All three samples (corresponding to 0 mM, 10 mM, and 100 mM NaCl treatments) exhibited diffraction patterns consistent with the hexagonal ZnO (zincite) phase (PDF# 01-078-2585), space group P6_3_mc. The major reflections matched well with standard ZnO peaks (Figure 5, diffractogram at the bottom). In terms of interplanar distances or spacings ($d_{hkl}$), the 0 mM diffractogram displayed a pure ZnO phase with only seven characteristic wurtzite peaks, showing the expected d-values (2.82, 2.60, 2.48, 1.91, 1.63, 1.48, and 1.38 Å) and no additional reflections. In the 10 mM diffractogram, in addition to these typical ZnO peaks, a single unidentified reflection was observed at d = 2.00 Å. In the 100 mM diffractogram, alongside the characteristic ZnO reflections, an additional non-characteristic and prominent peak was detected at d = 7.92 Å. (Figure 5).

### 2.4. Scanning Electron Microscopy (SEM) and Electron-Dispersive X-Ray Spectroscopy (EDS) of the Green Nanomaterial Derived from Duckweed

**Figure 6:**
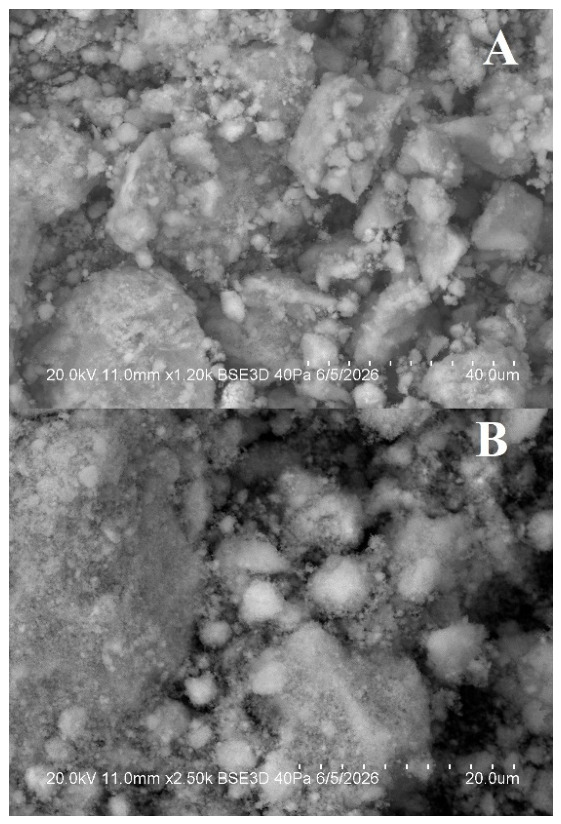
Representative SEM micrographs of ZnO nanoparticles synthesized using *Lemna minor* extracts. The particles formed irregular agglomerates consisting of finer substructures. Images were recorded using a Hitachi S-3700N microscope in variable-pressure mode with a BSE detector at an accelerating voltage of 20 kV. Scale bars: 40 μm (**A**) and 20 μm (**B**).

The surface morphology of the ZnO particles synthesized using *Lemna minor* extracts was investigated by scanning electron microscopy employing both backscattered electron (BSE) and secondary electron (SE) imaging modes. BSE micrographs revealed the formation of highly agglomerated particulate material consisting of irregular micron-sized aggregates distributed throughout the samples (Figure 6).

**Figure 7:**
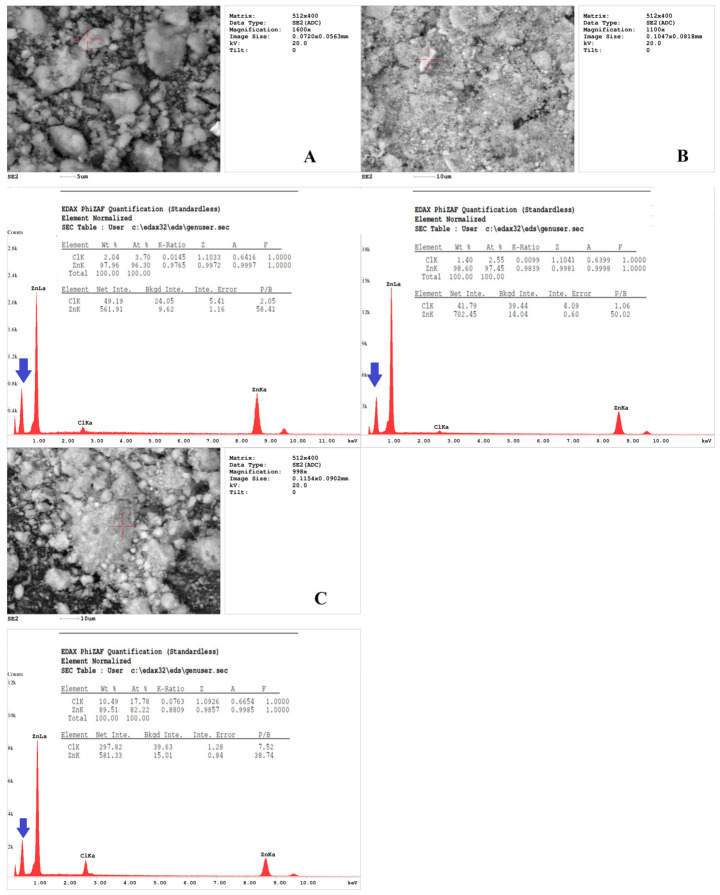
SEM micrographs and EDS spectra of ZnO nanoparticles synthesized using *Lemna minor* extracts obtained from plants grown under different salinity conditions: (**A**) 0 mM NaCl, (**B**) 10 mM NaCl, and (**C**) 100 mM NaCl. Images were acquired using a Hitachi S-3700N SEM operated at 20 kV in variable-pressure mode with a secondary electron (SE) detector, while semi-quantitative analysis was performed with EDS detectors. Blue arrows indicate signals attributable to oxygen. Scale bars: 5 μm (**A**) and 10 μm (**B**,**C**).

Higher-resolution SE micrographs provided additional information on surface morphology and showed that the larger aggregates were composed of finer submicron particles assembled into densely packed structures (Figure 7). The visible agglomerates ranged from approximately 2 to 30 µm, whereas the finer surface particles were mostly <1 µm. Nevertheless, all samples displayed pronounced aggregation, a characteristic commonly reported for plant-mediated ZnO nanoparticles. Due to the extensive agglomeration and the magnification range employed, individual primary nanoparticles could not be reliably distinguished or measured.

The elemental composition of the biosynthesized ZnO particles was investigated by energy-dispersive X-ray spectroscopy (EDS). The spectra of all samples were dominated by characteristic Zn signals, confirming zinc as the principal constituent of the synthesized material (Figure 7A–C). In addition to Zn, minor amounts of chlorine were detected in all samples, indicating the presence of residual chloride-containing species, most likely originating from the ZnCl_2_ precursor used during nanoparticle synthesis. Although lower-energy signals attributable to oxygen were consistently observed in the EDS spectra near ~0.5 keV, oxygen and carbon were not included in the quantitative analysis because reliable quantification of light elements is limited under the variable-pressure SEM-EDS conditions employed for the analysis of uncoated powder samples. Nevertheless, the combined SEM-EDS and XRD results consistently support the successful formation of ZnO as the predominant phase in all investigated samples.

### 2.5. (Poly)phenolic Content, DPPH Scavenging and Antimicrobial Activity of Duckweed-Derived ZnO NPs

The Folin–Ciocalteu (FC) test indicated the (poly)phenolic content of duckweed-derived ZnO NPs. This content increased with the concentration of the NP solution (Table 2). The highest phenolic content was measured for ZnO NPs at a 50 mg/mL final concentration. The DPPH scavenging activity also increased with the final concentration of NP suspension. The highest antioxidant activity was measured for ZnO NPs derived from duckweed exposed to 100 mM NaCl at an NP concentration corresponding to 50 mg/mL (Table 2).

To screen and compare the antimicrobial activity of nanoparticles at different concentrations and different duckweed treatments, the well diffusion test was performed (Table 2). There were no measurable halos in the agar plates streaked with *Serratia fonticola* A3-102/3. Also, this strain was absent in plates with 10 mg/mL Marocen as a positive control. Inhibition zones were measurable around wells with NP suspensions in agar plates streaked with *Staphylococcus haemolyticus* D4-2-100/1. These inhibition zones were at least 2.45 times narrower than the inhibition zones around wells with Marocen, which were estimated at (3.80 ± 0.21) cm. The antimicrobial activity of ZnO NP derived from stressed duckweed at 25 mg/mL was approximately 30% higher than that of ZnO NPs derived from mildly stressed or unstressed duckweed at 12.5 mg/mL (Table 2).

## 3. Discussion

### 3.1. Phytochemical Composition of Duckweed

Plants dynamically modify their chemical composition in response to different types of stress. In our previous research, we identified specific phenolic compounds such as luteolin 6,8-di-C-hexoside, p-hydroxybenzoic acid, caffeic acid, apigenin 6-C-(2″-pentosyl) hexoside and p-coumaric acid associated with endophytic colonization of duckweed by *Klebsiella oxytoca*, confirming that *L. minor* can remodel its phytochemistry in response to external (in this case, microbial) stimuli [15,37,38,39]. In addition, our recent research has shown that *Pseudomonas oryzihabitans* D1-104/3 and *P. gessardii* C31-106/3 differentially modulate the antioxidative response of duckweed to salt stress, leading to elevated levels of phenolic compounds [17]. The metabolic modulation of plants’ chemical composition confers multiple benefits to the plants, such as increased resistance to abiotic stress and defense against plant pathogens and predators. These effects may be translated into diverse biotechnological applications, e.g., medicinal use, feed for livestock and food for humans, or raw biomaterial production for bioethanol and biofertilizers, especially in the case of duckweeds [40,41,42,43,44]. Pagliuso et al. (2020) reported 16 flavonoids and their derivatives in the extracts of several duckweed species, most notably derivatives of chlorogenic acid, apigenin, and luteolin [43]. In this study of a single strain of *L. minor*, we identified six: a vitexin isomer, vitexin 2″-O-xyloside, schaftoside isomers 1 and 2, luteolin-7-O-glucoside, apigenin and apigenin 7-O-hexoside. Interestingly, C-glycosides were the most prevalent form of flavones, similarly to our study, where luteolin-O-glucoside and schaftoside isomers were reported. Moreover, the presence of uralenneoside and feruloylmalic acid reported here is a rare and unique finding in *Lemna minor* research. This highlights the metabolic plasticity and differences between phylogenetically close duckweed species and the need to characterize the phenolic content of multiple strains of *L. minor* and of other related duckweed species.

Among the few studies analyzing the connection between salt stress and green nanoparticle synthesis, Gupta et al. (2024) reported that 75 mM NaCl triggered a qualitative shift in the phytochemical composition of the giant duckweed (*Spirodela polyrhiza*), marked by the accumulation of flavonoid subset, namely flavones and their glycosides (orientin, genistin, azelaic acid, sinapic acid, quercetin, apigetrin, vitexin, and naringin) as the key metabolites for the nucleation and stabilization of nanoparticles [9]. In this study, based on the growing contribution of PC1 to total variability, we found that the phenolic profile of *Lemna minor* is strongly associated with salt stress, with distinct metabolic shifts observed across 0, 10, and 100 mM NaCl treatments, which is aligned with our previous work [17]. Moreover, based on the compounds’ influence on PC1 and PC2 constructions, it can be concluded that every treatment has a specific phenolic footprint. The following flavonoids and hydroxycinnamic acids responded to increasing salt stress: schaftoside isomers 1 and 2, luteolin-7-o-glucoside, feruloylmalic acid, vitexin 2″-O-xyloside, uralenneoside, sinapic acid, 1-O-sinapoyl-β-D-glucose, dihydroxybenzoic acid hexoside, caffeic acid hexoside, and coumaric acid. Similarly, as in Gupta et al., glycosylated flavones (schaftoside isomers, luteolin, vitexin, and uralenneoside derivatives) also accumulated with increasing salt stress [9].

Therefore, it would be of research interest to test multiple duckweed strains and species, as duckweeds (family Lemnaceae) exhibit extraordinary metabolic plasticity and variety, with the added advantage of rapid biomass doubling and minimal growth requirements compared to some other conventionally used plant species. In the future, special attention should be given to phenylpropanoid and flavonoid pathways, as they are key determinants of the physicochemical properties of biosynthesized nanoparticles.

### 3.2. UV–Vis Spectroscopic Properties of Duckweed-Derived ZnO Nanoparticles

The UV–Vis absorption spectra of nanoparticles synthesized from *Lemna minor* extracts grown under different NaCl concentrations provide further insight into the influence of metabolic plasticity on nanoparticle formation.

All samples exhibited distinct peaks around 360 nm, indicative of small, uniformly distributed nanoparticles, as supported by relevant literature [1,45,46]. This also corresponds well with the Orbitrap analysis, which revealed elevated levels of key reducing and stabilizing phenolics such as caffeic acid hexoside and ferulic acid, both known to accelerate metal ion reduction and promote controlled nucleation [3,47].

The almost fixed position of the characteristic 360 nm absorbance maximum across all spectra confirmed the presence of the same type of ZnO nanoparticles in all samples, while the differences in intensity hinted at different reaction yields and colloidal effects (most probably scattering and aggregation), as well as interactions between nanoparticles and other components in the suspension (e.g., unbound phytochemicals). This agrees with Gupta et al. (2024), who observed diverse, polydispersed, and rosette-like zinc nanostructures synthesized from the extract of duckweed grown at 75 mM NaCl compared to control plants [9]. However, whether similar structures are indeed present in the ZnO NP samples in this study remains to be seen.

The apparent *E*_g_ values were consistent with those reported for similar green-synthesized ZnO nanomaterials [48]. Interestingly, Rafique et al. also reported band gaps in the 2.20–2.60 eV range, where band gaps decreased as extract volume increased [1]. The *E*_g_ differences between samples might also reflect this effect. Moreover, the apparent band gap shrinking compared to bulk ZnO (3.3 eV) may be associated with differences in defect concentration, lattice disorder, surface states, and/or the presence of phytochemical species adsorbed on the nanoparticle surface. This interpretation is indirectly supported by the fluorescence-dominated Raman spectra, which hint at the presence of surface-associated organic compounds, as well as by SEM–EDS analysis confirming Zn-rich particulate materials with prominent peaks corresponding to O Kα emissions and minor variations in residual chloride content among the samples. Further investigations employing photoluminescence spectroscopy, X-ray photoelectron spectroscopy, or advanced defect characterization techniques would be required to elucidate the origin of the apparent band gap reduction compared to bulk ZnO.

### 3.3. The Polycrystalline Nature of Duckweed-Derived ZnO Green Nanoparticles

The X-ray diffraction (XRD) results provide compelling evidence for the successful synthesis of ZnO nanoparticles under all tested salinity conditions, as confirmed by the presence of characteristic ZnO diffraction peaks. However, variations in the diffraction patterns across different salinity levels also suggest that the salinity exposure of the duckweed extract had an influence on the phase purity and possible secondary phase formation during nanoparticle synthesis.

At 0 mM NaCl, the XRD profile exhibited a clean and sharp pattern corresponding exclusively to the hexagonal wurtzite ZnO structure, with no detectable secondary reflections. This indicates that the extract obtained from unstressed duckweed acts as an effective biological reducing and stabilizing agent, capable of facilitating the formation of pure ZnO nanoparticles. The absence of any impurity peaks suggested minimal interference from plant metabolites, precursor residues or decomposition products in the synthesis process.

In contrast, the XRD pattern of the 10 mM NaCl sample showed a minor, yet notable, unidentified diffraction peak at d = 2.00 Å. While the dominant peaks still matched the ZnO phase, the emergence of this additional reflection implies the presence of a secondary crystalline phase, possibly a minor chloride crystalline residue stemming from either the precursor or NaCl treatment. This could tentatively be attributed to subtle alterations in the biochemical composition of the duckweed extract induced by moderate salt stress.

However, the most pronounced deviation from the expected diffraction pattern was observed in the 100 mM NaCl sample, where, in addition to typical ZnO peaks, a strong reflection at d = 7.92 Å was recorded. The intense abiotic stress at 100 mM NaCl likely prompted a biochemical shift in the duckweed extract, enhancing the complexity of interactions between Zn^2+^ ions and organic constituents during nanoparticle formation. In other studies, similar additional signals corresponded to layered zinc hydroxide complexes, the by-products of nanoparticle synthesis [13,49]. The isolated non-ZnO reflections differed among the samples, suggesting that the minor secondary crystalline components were not identical under all synthesis conditions. The absence of the d ≈ 2.00 Å reflection in the 100 mM sample indicates that this phase was either absent, below the XRD detection limit, or replaced by another minor phase responsible for the low-angle d ≈ 7.92 Å reflection.

Raman spectroscopy was used as a complementary technique to probe vibrational properties and structural disorder of the synthesized ZnO nanoparticles. However, due to the intense fluorescence of ZnO NPs, Raman spectra remained broad and featureless, without clearly resolved ZnO Raman bands (Supplementary Figure S2). This behavior is consistent with the observation that Raman spectra of plant-extract-derived nanomaterials are sensitive to fluorescence originating from surface-bound phytochemicals [2]. For wurtzite ZnO, the E_2_(high) mode near 437–440 cm^−1^ is generally associated with crystalline lattice order, whereas LO-region bands around 590–660 cm^−1^ are commonly linked to intrinsic defects such as oxygen vacancies and zinc interstitials [50,51].

In our study, Raman spectra were dominated by a strong fluorescence background, resulting in broad and poorly resolved spectral features (Supplementary Figure S2.1). Weak contributions were observed within the spectral regions commonly associated with the E_2_(high) and LO modes of wurtzite ZnO; however, their assignment remains tentative due to the low signal-to-background ratio and extensive band broadening. Such broadening may arise from nanoscale disorder, defect-related states, and interactions between the ZnO surface and phytochemical capping agents. The progressive increase in fluorescence intensity from 0 to 10 and 100 mM samples further supports an increasing contribution of surface-associated phytochemicals, in agreement with the Orbitrap metabolomic analysis.

Therefore, XRD and SEM–EDS were used as the primary evidence for ZnO phase formation, while Raman spectroscopy was considered complementary and mainly informative regarding the surface-modified, defect-rich nature of the green-synthesized particles. Collectively, the Orbitrap, UV–Vis, XRD, SEM–EDS, and Raman results suggest that salinity predominantly influenced the surface chemistry of the green-synthesized ZnO nanoparticles through modulation of phytochemical composition, while the underlying ZnO crystalline structure remained largely preserved.

Overall, these observations highlight the dual role of the duckweed extract as both a reducing/stabilizing agent and a modulator of the final nanoparticle structure, depending on its physiological state under salinity stress. The emergence of secondary reflections in stressed samples suggests that stress-induced phytochemicals can alter the crystallization pathway, leading to hybrid or composite nanostructures, which may explain the additional signals in diffractograms. The differences between diffractograms can, hypothetically, be the result of distinctive ratios of phenolic compounds during treatments, a phenomenon underlined by PCA. The positive FC test hinted at the presence of (poly)phenolic compounds in green ZnO NPs. Interestingly, Gupta et al. (2024) studied the structure of nanoparticles derived from stressed giant duckweed, grown at 75 mM NaCl and reported broadly similar observations to those in our paper: accumulation of flavonoids, wurtzite ZnO resulting from green synthesis, and micronic agglomerates of nanomaterial with finer submicronic structures [9]. To the best of our knowledge, this study is the first to compare diffraction patterns, EDS-SEM, and Raman spectra of nanoparticles derived from the extract of duckweed grown under different physiological conditions. These observations highlight the potential of manipulating plant stress responses to fine-tune the properties of green-synthesized nanomaterials. Although the combined Orbitrap, UV–Vis, XRD, SEM–EDS, and Raman analyses provide valuable insight into the properties of the green-synthesized ZnO nanoparticles, further characterization by TEM, XPS, FTIR, and BET analysis would be beneficial for a more comprehensive understanding of nanoparticle morphology, surface chemistry, porosity, and capping mechanisms. These investigations will be the subject of future studies.

### 3.4. Biological Activity of Duckweed-Derived ZnO Nanoparticles

The antioxidant and antimicrobial performance of duckweed-derived ZnO nanoparticles was significantly influenced by both salt treatment and nanoparticle concentration. Total phenolic content (TPC) increased notably with higher NP concentrations, particularly under moderate (10 mM) and high (100 mM) NaCl stress, hinting at the role of salt stress in enhancing phenolic components in the structure of duckweed-derived ZnO NPs. The highest TPC (66.79 μM GAE/g) was observed at 50 mg/mL ZnO NPs in duckweed exposed to 10 mM NaCl, suggesting this condition effectively promotes (poly)phenol incorporation during synthesis, which is aligned with the shifts in phenolic profiles revealed by Orbitrap analysis. Similar results were reported in other studies [1,2,46]. Overall, these results hint at the important role of phenolics in the structural organization and biofunctionality of duckweed-derived ZnO nanoparticles.

Antioxidant activity, as measured by DPPH scavenging, showed a sharp increase under 100 mM NaCl at 50 mg/mL, reaching 55.01% and far surpassing other treatments. This was to be expected from our previous antioxidant activity study of pure duckweed extract [17]. Again, this indicated that severe salt stress may trigger the accumulation of potent antioxidant compounds that are retained in the nanoparticle matrix.

Antimicrobial activity, as reflected by inhibition zones, also improved, albeit modestly, with both NP concentration and salt stress, with the largest zones (1.55 cm) observed for NPs synthesized under 10 and 100 mM NaCl at 25 mg/mL. An interesting observation was made regarding the biological specificity of ZnO NPs in this study, where the ZnO NPs exerted inhibitory effects only against the Gram-positive strain and apparently had no effect on the Gram-negative one. In similar studies, green NPs exerted antimicrobial effects against both Gram-positive and -negative bacteria equally, or more against Gram-positive bacteria while Gram-negative appeared more resistant [2,9,16,19,52]. For instance, Shukla et al. (2025) report equal inhibitory zones of ZnO NPs against *Bacillus subtilis* (a Gram-positive bacterium) and *Salmonella typhi* (a Gram-negative bacterium) [1]. The inhibitory zones in this and similar studies were comparable to those measured in this study, in the range of 0.9–1.5 cm [1]. This underlines the biological specificity of ZnO NPs, which can at least in part be explained by the universal differences in the structure of the bacterial cell wall of Gram-positive versus Gram-negative bacteria, as well as by the resistance mechanisms of individual bacterial strains tested herein [2,5]. The effect of NP concentration was also reported and hinted that higher NP concentrations may effectively reduce their mobility and diffusion through the agar, mostly due to aggregation [29]. At higher concentrations, NPs tend to form clusters that may hinder their mobility through the porous structure of agar, which reduces their effective antimicrobial potency even though their biological activity remains unchanged.

Together, these results suggest that salt stress enhances the biofunctionalization of ZnO NPs by increasing the availability and activity of phenolic compounds, particularly at higher NP concentrations, thus improving their antioxidant and antimicrobial efficacy. The green ZnO NPs synthesized in this study may have some practical applications, such as, for instance, topical antimicrobial ointments against infections caused by Gram-positive bacteria or as a treatment against Gram-positive bacterial phytopathogens. Moreover, the generally low toxicity and environmental impact of zinc in this hypothetical formulation sets it apart from other, more commonly used silver-based nanomaterials [27,29]. However, stability testing of the nanoparticle suspensions, including shelf-life, dispersion stability, and retention of functional properties over time, was not conducted in this study due to the preliminary nature of the work. Given the importance of these parameters for assessing real-world applicability, their evaluation remains a priority for future research.

Finally, as duckweed biomass is rapidly doubled, the green synthesis of nanomaterials in this case would be a particularly affordable and self-sustainable process. Thus, the green synthesis of antimicrobial ZnO NPs based on duckweed biomass connects circular economy with sustainable biomedicine, revalorizing duckweed biomass which is, as it currently stands, globally underutilized.

## 4. Materials and Methods

### 4.1. Duckweed (L. minor) Growth Conditions

Duckweed was grown under the same conditions as described before [17]. In brief, surface-sterilized duckweed was maintained in stock cultures with complete Murashige–Skoog medium supplemented with 3% sucrose at 24 ± 2 °C under fluorescent light of 40 µmoL m^−2^ s^−1^ with a 16 h light/8 h dark photoperiod. For the salt exposure experiments, 150–300 individual fronds were picked and transferred to MS medium without vitamins or sucrose (in further text: MS medium). Fronds were acclimatized and then transferred to fresh sterile MS medium in three groups based on supplementation with sodium chloride (NaCl)—control (0 mM), 10 mM, and 100 mM—for 14 days.

### 4.2. Phytochemical Characterization of Phenolic Compounds in Duckweed Exposed to Salt Stress

#### 4.2.1. Sample Preparation

To identify the presence of different metabolites in the extracts of duckweed exposed to increasing salt stress, the fresh weight of duckweed was collected after 14 days of cultivation and immediately ground to fine powder in liquid nitrogen as described in our previous work [17]. The ensuing extracts were sonicated for 1 h with cooling in ice and centrifuged, and the supernatants were filtered through a syringe with a filter pore size of 0.2 µm immediately before HPLC–Orbitrap-MS analysis.

#### 4.2.2. HPLC Parameters

An analytical Hypersil GOLD™ C18 column (Thermo Fisher Scientific, Waltham, MA, USA; 50 × 2.1 mm, 1.9 μm particle size) was used. Injection volume was 5 μL with a constant flow rate of 300 μL/min. Mobile phases: ultra-pure water with 0.1% formic acid (A) and acetonitrile with 0.1% formic acid (B) were eluted in the following ratios: 5% B at minute 1; 5–95% B from minute 1 to 10; 95% B from minute 10 to 12; and 5% B until minute 15.

#### 4.2.3. Orbitrap Parameters

The Orbitrap Exploris 120 mass spectrometer (Thermo Fisher Scientific) was equipped with an electrospray ionization (ESI) source in negative ionization mode. Full MS scans were performed in the 100–1500 *m*/*z* range with a resolution of 60,000 FWHM. Data-dependent acquisition (DDA) MS2 experiments were conducted at a resolution of 15,000 FWHM with a normalized collision energy (CID) of 35%. Dynamic exclusion was set to 10 s, with exclusion after two scans, and the intensity threshold was set at 1 × 10^5^.

### 4.3. Green Synthesis of ZnO Duckweed-Derived Nanoparticles

Duckweed was collected and oven-dried at 60 °C until a constant dry weight (DW) was achieved. The DW was ground in liquid nitrogen with a mortar and pestle. Ethanol solution (80%) was added as an extractant in a 1:20 ratio (*v*:*v*). The ensuing extract was heated at 60 °C for 20 min and gently mixed. After cooling, the extract was used in green synthesis according to [1]. Briefly, a 0.5 M solution of zinc chloride (ZnCl_2_) was prepared. The plant extract was added dropwise to the ZnCl_2_ solution at a 1:2.5 ratio (*v*:*v*). The pH of the mixture was adjusted to 9.0 by the gradual addition of 1 M NaOH solution. The flask was heated to 70 °C on a magnetic stirrer with a constant stirring and heating option. The appearance of a pale yellow to pale yellow-green precipitate indicated the formation of ZnO nanoparticles.

#### Post-Processing

The precipitate from the previous step was sequentially centrifuged and washed 3 times and then sonicated for 20 min at 40% frequency in an ultrasonic water bath (Bandelin Sonorex Digiplus, Bandelin GmbH, Berlin, Germany). The resulting sediment was oven-dried at +60 °C for 15 h in glass Petri dishes. After the sediment was dried, thermal processing was performed at 150 °C for 2 h in a drying oven (Memmert GmbH, Schwabach, Germany). The dry sediment was then ground to a fine powder with a mortar and pestle and kept in airtight containers in the dark for further analysis.

### 4.4. X-Ray Diffraction (XRD) of Powdered ZnO Duckweed-Derived Nanoparticles

To determine the phase purity and crystallinity of synthesized nanoparticles, X-ray diffraction (XRD) on powdered material was performed on a Rigaku SmartLab diffractometer (Rigaku Holdings Corporation, Tokyo, Japan) at the Laboratory for Crystallography of the Faculty of Mining and Geology, University of Belgrade. Zinc oxide nanoparticles were synthesized using extracts of duckweed (*Lemna minor*) cultivated under varying NaCl concentrations: 0 mM, 10 mM, and 100 mM. The crystalline phases of the resulting powders were analyzed by powder X-ray diffraction (PXRD) using CuKα roentgen radiation (λ = 1.54178 Å) emitted from a copper anticathode over a 2θ range of 2–70°, with a step size of 0.01° and a scan rate of 5°/min. The working voltage (U) was 40 kV, with the current intensity (I) of 30 mA. The obtained data of diffraction maxima 2Θ (°), the interplanar distances *d_hkl_* (Å) for all three *hkl* reflections, and the appropriate relative intensities *I*/*I_max_* were presented graphically. The crystalline phases were identified based on the acquired values of *I*/*I_max_*, the interplanar distances *d* and the comparison with literature data and the ICDD database.

### 4.5. Scanning Electron Microscopy and Energy-Dispersive X-Ray Spectroscopy (SEM–EDS)

The morphology and elemental composition of the synthesized ZnO samples were examined using a Hitachi S-3700N scanning electron microscope (Hitachi High-Technologies, Tokyo, Japan) equipped with a secondary electron (SE), a backscattered electron (BSE), and energy-dispersive X-ray (EDS) detectors. Elemental analysis was performed using a Quantax 200 EDS system (Bruker, Mannheim, Germany) with an energy resolution of 123 eV at Mn Kα. SEM observations were conducted under variable-pressure conditions to minimize the charging effects of the non-conductive powder samples. The EDS measurements were used for the semi-quantitative determination of the elemental composition and distribution within the analyzed samples.

### 4.6. UV–Vis Characterization of ZnO Duckweed-Derived Nanoparticles

The nanomaterial was resuspended in double-distilled water. Absorbance was measured across the wavelength range of 300–600 nm with a UV–Vis Agilent 8453 spectrophotometer (Agilent Technologies, Santa Clara, CA, USA) in a 1400 µL Suprasil quartz cuvette (Hellma GmbH & Co. KG, Müllheim, Germany). Multiple spectra (at least 3 per sample) were read, and optical characteristics were monitored. From the UV–Vis spectra, the Tauc plots were deduced. The Tauc method is based on the equation [51]:

(1) $${\left(\alpha \times h\upsilon \right)}^{\frac{1}{\gamma }}=B\times (h\upsilon -E_{g})$$

where h is the Planck constant, υ is the photon’s frequency, *E*_g_ is the apparent band gap energy, and B is a constant. The γ factor depends on the nature of the electron transition and is equal to 1/2 or 2 for the direct and indirect transition band gaps, respectively.

### 4.7. Total Polyphenol Content (TPC) and DPPH Method (Antioxidant Capacity) on ZnO Duckweed-Derived Nanoparticles

The methods outlined in our previous work were used to determine the total polyphenol content (TPC) and antioxidant capacity of ZnO nanoparticles [17]. Briefly, the quantification of TPC was conducted based on Folin–Ciocalteu principles, whereas antioxidant capacity was determined with the use of the DPPH (1,1-diphenyl-2-picrylhydrazyl) technique. For TPC, the absorbance was measured at 765 nm. Gallic acid was used as a standard. For DPPH, the same nanoparticle solutions used for the FC test were mixed with DPPH reagent and dissolved in methanol. The samples were incubated at room temperature in the dark alongside controls (reaction mixture without DPPH, to account for the relatively high absorbance of nanoparticles around 520 nm), and absorbance was measured at 520 nm. The scavenging ability of DPPH radical was calculated as:

Inhibition (%) = 1 − (Abs_sample_ − Abs_control_) × 100 (2)

### 4.8. Antimicrobial Activity of ZnO Duckweed-Derived Nanoparticles (Well Diffusion Test)

For the well diffusion test, fresh overnight cultures of bacterial strains *Staphylococcus haemolyticus* D4-2-100/1 and *Serratia fonticola* A3-102/3 from our previous work were used for the test and cultivated as reported before [17,53]. The wells were created with sterile 1 mL pipette tips and filled with 50 µL of gradually increasing concentrations of ZnO NP solutions (12.5, 25, and 50 mg/mL). Sterile double-distilled water was used as a negative control. Ciprofloxacin (Marocen, Hemofarm, Vršac, Serbia) was used as a positive control. The plates were incubated at +37 °C for 24 h. The halo zones were measured in *ImageJ* Version 1.53K [54] as an indication of the inhibitory effect of the antibiotic and the nanomaterial.

### 4.9. Statistical Analysis and Graphic Presentation of Results

A one-way ANOVA test (*p* < 0.05) was performed in STATISTICA software, Version 7, with a post hoc Fisher LSD test for antimicrobial and DPPH activity and the FC test. For the principal component analysis (PCA) and the heatmap, the open-source statistical software R, version 4.5.1 [55], was used. PCA and construction of a vector plot of variables were performed using the FactoMineR package [56]. Additionally, the Factoextra package was used for data clustering and biplot representation of data [57,58]. Heatmap analysis was performed with the ComplexHeatmap package, as log10 values of means from each treatment and result were clustered based on change in parameter values [59]. For distance clustering, the “maximum distance” method was used, in which the distance between two clusters is determined by finding the single pair of points (one from each cluster) that are farthest apart and using that distance as the measure.

## 5. Conclusions

This observational study provides preliminary evidence that the physiological state of *Lemna minor* during cultivation may influence both the phytochemical composition of plant extracts and certain properties of green-synthesized ZnO nanoparticles. Orbitrap analysis revealed salinity-dependent shifts in the metabolite profile of duckweed, including increased abundance of several flavonoids, glycosylated flavones, and hydroxycinnamic acid derivatives under salt stress. These changes were accompanied by differences in the characteristics of the resulting nanomaterials.

UV–Vis spectroscopy, PXRD, SEM–EDS, and Raman spectroscopy collectively support the formation of ZnO nanoparticles using duckweed extracts cultivated under 0, 10, and 100 mM NaCl. PXRD analysis indicated the predominance of crystalline hexagonal wurtzite ZnO in all samples, while SEM observations revealed micron-scale agglomerates composed of finer submicron structures. EDS spectra confirmed Zn-rich materials with oxygen-associated signals consistent with ZnO formation. Raman spectra were dominated by fluorescence, which increased with salinity treatment and may reflect differences in surface-associated phytochemical species among the samples. Taken together, the available data tentatively suggest that salinity had a limited effect on the crystalline structure of the nanoparticles but may have influenced their surface chemistry through changes in extract composition. However, the extent to which these phytochemical differences contribute to the observed antioxidant and antimicrobial activities remains unclear. Additional studies are needed to distinguish between the effects of nanoparticle structure, surface-bound phytochemicals, and potential synergistic interactions between them.

The biological properties of the nanoparticles also appeared to vary with the cultivation conditions of the source biomass. Nanoparticles synthesized using extracts from salt-stressed duckweed exhibited higher total phenolic content, antioxidant capacity, and antimicrobial activity against *Staphylococcus haemolyticus*. While the underlying mechanisms remain to be fully elucidated, these observations are consistent with the possibility that salinity-induced metabolic changes in duckweed contributed to the biological properties of the resulting nanomaterials.

Overall, the results suggest that environmental manipulation of plant biomass may represent a promising approach for influencing green nanoparticle synthesis. By integrating plant stress physiology, metabolomic profiling, and nanomaterial characterization, this study highlights potential links between cultivation conditions and nanoparticle properties, while also emphasizing the need for further investigations to establish the mechanistic basis of these relationships.

## Figures and Tables

**Table 1:** Qualitative results of Orbitrap analysis ^1^.

#	Compound	RT	Formula, [M-H]^−^	Calculated Mass, [M-H]^−^	Exact Mass, [M-H]^−^	ppm	MS2 Fragments	NaCl conc. (mM)/Biological Replicates								
								0			10			100		
								1	2	3	1	2	3	1	2	3
1	Trihydroxybenzoic acid 1	0.51	C_7_H_5_O_5_	169.014247	169.014584	−1.99	123.54(12), **125.02**(100), 169.01(40)	-	+	-	-	-	-	-	-	-
2	Dihydroxybenzoic acid hexoside	0.71	C_13_H_15_O_9_	315.072156	315.0725586	−1.28	108.02(43), **152.01**(100), 153.02(53), 315.071(50),	+	+	-	+	+	-	+	+	-
3	Caffeic acid hexoside	1.24	C_15_H_17_O_9_	341.087806	341.0876429	0.48	59.01(4), 135.04(4), **161.02**(100), 179.03 (13)	+	+	+	+	+	+	+	-	+
4	Uralenneoside	1.25	C_12_H_13_O_8_	285.061591	285.0621058	−1.81	108.02(54), **152.01**(100), 153.02(58), 285.06 (48)	+	+	+	+	+	+	+	+	-
5	Syringic Acid Hexoside	1.25	C_15_H_19_O_10_	359.098371	359.0981958	0.49	**59.01**(100), 89.02 (90), 101.02(46), 119.03(37), 197.05(95)	+	-	+	-	-	-	+	-	-
**6** [33]	p-Coumaroylglucose	4.26	C_15_H_17_O_8_	325.092891	325.0935204	−1.94	59.01(4), 119.05(4), **145.03**(100), 163.04 (10)	+	-	-	+	+	-	+	+	-
**7** [33]	1-O-Sinapoyl-β-D-glucose	4.63	C_17_H_21_O_10_	385.114021	385.1138538	0.43	59.01(9), 190.03(17), **205.05**(100), 223.05(15)	+	-	+	+	+	+	-	+	+
8	Hydroxybenzoic acid 1	4.69	C_7_H_5_O_3_	137.024418	137.0246764	−1.89	93.03(50), **136.86**(100), 137.02(62)	+	-	-	+	+	-	+	+	-
9	Hydroxybenzoic acid 2	4.94	C_7_H_5_O_3_	137.024418	137.0247078	−2.11	93.03(71), **136.86**(100), 137.02(88)	-	-	-	-	-	-	-	-	+
10	Sinapic acid	5.01	C_11_H_11_O_5_	223.061197	223.0614305	−1.05	149.02(85), **164.05**(100), 208.04(16)	+	-	+	-	+	+	+	+	+
11	Caffeoylglucaric acid	5.16	C_15_H_15_O_11_	371.061985	371.0616524	0.9	85.03(30), **111.01**(100), 129.02(12), 154.99(15)	+	-	-	+	+	-	+	+	-
12	Esculetin	5.20	C_9_H_5_O_4_	177.019332	177.019521	−1.07	105.03(32), **133.02985**(100)	+	-	+	+	-	+	-	+	+
13	Fertraric acid	5.34	C_14_H_13_O_9_	325.056506	325.0567898	−0.87	115.00(16), **133.01**(100)	+	+	-	+	-	-	-	+	-
14	Cinnamic acid	5.42	C_9_H_7_O_2_	147.045153	147.0453699	−1.47	**102.95**(100)	+	+	-	+	+	+	+	+	-
15	Caffeic acid	5.47	C_9_H_7_O_4_	179.034982	179.0351828	−1.12	134.99(22) **135.04**(100), 179.04(23)	-	-	+	-	-	+	+	+	+
16	Coumaric acid	5.66	C_9_H_7_O_3_	163.040068	163.0402948	−1.39	**119.05**(100), 163.04(16)	+	+	+	+	+	+	+	+	-
17	Feruloylmalic acid	5.87	C_14_H_13_O_8_	309.061591	309.0620012	−1.33	71.01(20), 115.00(11), 134.04(42), 149.06(14), **193.05**(100)	+	+	+	+	+	+	+	+	-
18	Benzoic acid	5.92	C_7_H_5_O_2_	121.029503	121.0297292	−1.87	66.00(13), **121.03**(100)	+	+	+	+	+	+	+	+	+
19	Hydroxybenzoic acid 3	6.10	C_7_H_5_O_3_	137.024418	137.0246535	−1.72	93.03(77), **136.86**(100), 137.02(87)	+	+	-	+	+	-	+	+	-
20	Ferulic acid	6.33	C_10_H_9_O_4_	193.050632	193.0508996	−1.39	134.04(36), **193.05**(100)	+	+	-	+	+	+	+	+	-
**Flavonoids and their glycosides**																
**21** [34]	Vitexin 2″-O-xyloside	5.19	C_26_H_27_O_14_	563.140629	563.1413818	−1.34	**353.07**(100), 383.08(71), 563.14(30)	-	+	+	-	-	+	-	-	+
**22** [35]	Schaftoside isomer 1	5.41	C_26_H_27_O_14_	563.140629	563.1410354	−0.72	**353.07**(100), 383.08(73), 443.10(74), 473.11(54), 563.14(67)	+	+	+	+	+	+	+	+	+
**23** [36]	Schaftoside isomer 2	5.54	C_26_H_27_O_14_	563.140629	563.1409174	−0.51	**353.07**(100), 383.08(76), 443.10(57), 473.11(48), 563.14(58)	+	+	-	+	+	-	+	+	-
24	Vitexin isomer	5.76	C_21_H_19_O_10_	431.098371	431.097928	1.03	59.01(32), **311.06**(100), 341.07(35)	+	+	-	+	+	-	+	+	-
25	Luteolin-7-o-glucoside	5.86	C_21_H_19_O_11_	447.093285	447.093126	0.36	284.03(18), **285.04**(100), 447.09(25)	+	+	+	+	+	+	+	+	+
26	Apigenin 7-O-hexoside	6.17	C_21_H_19_O_10_	431.098371	431.0975957	1.8	59.01(16), 268.04(66), 269.04(54), **431.10**(100)	+	+	-	+	+	-	+	+	-
27	Apigenin	6.50	C_15_H_9_O_5_	269.045547	269.0459727	−1.58	151.00(2), **269.05**(100), 270.05(2)	+	+	+	+	+	+	+	+	+
**Iridoid glycosides**																
28	Geniposidic acid	6.46	C_16_H_21_O_10_	373.114021	373.1141707	−0.4	59.01(38), 85.03(22), **111.01**(100)	-	-	-	+	-	-	+	+	-
**Other compounds**																
29	Piperonal	5.88	C_8_H_5_O_3_	149.024418	149.0245845	−1.12	93.03(12), **121.03**(100), 149.02(33)	+	+	-	+	+	-	+	+	-
30	Jasmonic acid	8.98	C_12_H_17_O_3_	209.118318	209.1186386	−1.53	125.10(26), 127.90(24), 209.10(19), **209.12**(100),	-	-	-	-	+	-	+	+	-

^1^ Compounds are presented with their retention times (RT), molecular formulas, mass accuracy (ppm), and peaks corresponding to masses of molecular fragments in the electric field. Mass accuracy between −5 and +5 was considered acceptable. The presence of a compound in a biological replicate was marked by: +; the absence by: -.

**Table 2:** Total phenolic content (TPC), DPPH scavenging and antimicrobial activity (inhibition zone) of NP solutions.

Salt Treatment, Final Concentration of NPs	TPC (GAE μM/g)	Scavenging Activity (%)	Inhibition Zone (cm)
0 mM NaCl, 12.5 mg/mL	0.34 ± 0.01 ^d^	8.47 ± 1.11 ^c^	1.17 ± 0.05 ^c^
0 mM NaCl, 25 mg/mL	38.67 ± 0.12 ^b^	11.51 ± 1.81 ^bc^	1.45 ± 0.02 ^ab^
0 mM NaCl, 50 mg/mL	**61.91 ± 0.36 ^a^**	15.31 ± 3.58 ^bc^	1.36 ± 1.40 ^ab^
10 mM NaCl, 12.5 mg/mL	17.95 ± 0.04 ^c^	9.45 ± 0.39 ^c^	1.30 ± 0.07 ^bc^
10 mM NaCl, 25 mg/mL	40.06 ± 0.33 ^b^	11.69 ± 0.57 ^bc^	**1.55 ± 0.13 ^a^**
10 mM NaCl, 50 mg/mL	**66.79 ± 0.15 ^a^**	11.82 ± 1.63 ^bc^	1.44 ± 0.03 ^ab^
100 mM NaCl, 12.5 mg/mL	0.48 ± 0.01 ^d^	9.66 ± 0.93 ^c^	1.40 ± 0.10 ^ab^
100 mM NaCl, 25 mg/mL	54.38 ± 0.09 ^b^	25.89 ± 13.89 ^b^	**1.55 ± 0.05 ^a^**
100 mM NaCl, 50 mg/mL	**59.97 ± 0.18 ^a^**	**55.01 ± 0.21 ^a^**	1.50 ± 0.05 ^ab^

Values represent mean ± S.E. Different letters within columns indicate significant differences between treatments at *p* ≤ 0.05. Maximum values are bold.

## Data Availability

The data presented in this study are available upon request from the corresponding author due to technical reasons.

## Supplementary Materials

The following supporting information can be downloaded at: https://www.mdpi.com/article/10.3390/molecules31132326/s1, Figure S1A–D: Histograms representing influence of phenolic compounds on PC1 and PC2, which form coordinates for principal component analysis (PCA) plot of 30 phenolic compounds isolated from duckweed grown at 0 mM NaCl, 10 mM NaCl, and 100 mM NaCl (A); at 0 mM (B); at 10 mM (C); at 100 mM (D); Figure S2: Raman spectroscopy of three green ZnO nanomaterials.

## Abbreviations

The following abbreviations are used in this manuscript:

ZnO NPs	Zinc-oxide nanoparticles
FTIR	Fourier transform infrared spectroscopy
SEM/TEM	Scanning electron microscopy/ transmission electron microscopy
EDS	Energy-dispersive X-ray spectroscopy
XPS	X-ray photoelectron spectroscopy
BET	Brunauer–Emmett–Teller analysis
