# Supplementary material for: The Role of Phenolic Profile of Salt-Stressed Duckweed (Lemna minor) in Synthesis and Biological Activity of Green ZnO Nanoparticles
Source: Molecules. 2026 Jul 2;31(13):2326. doi: 10.3390/molecules31132326 (PMC13362555; doi:10.3390/molecules31132326)
Supplement: Supplementary file 1 [file molecules-31-02326-s001.zip › molecules-4089079-supplementary.pdf]

Supplementary Figure S1. Contribution of the variables.

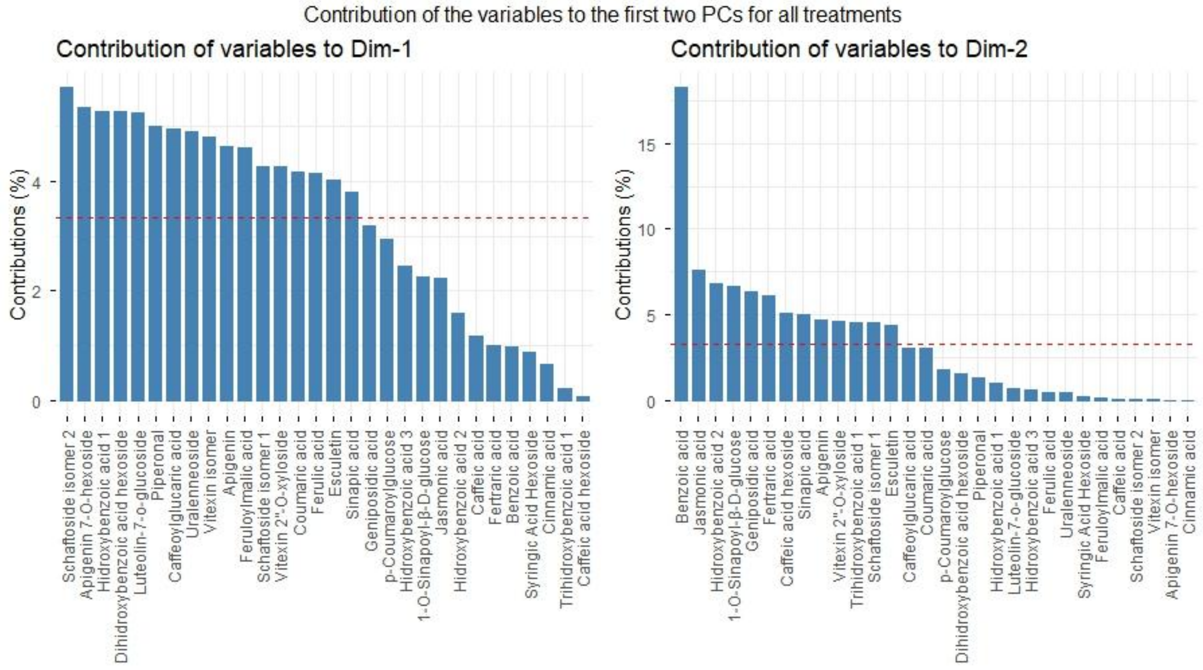

Figure S1. A: Contribution of the variables to the principal components for all treatments.

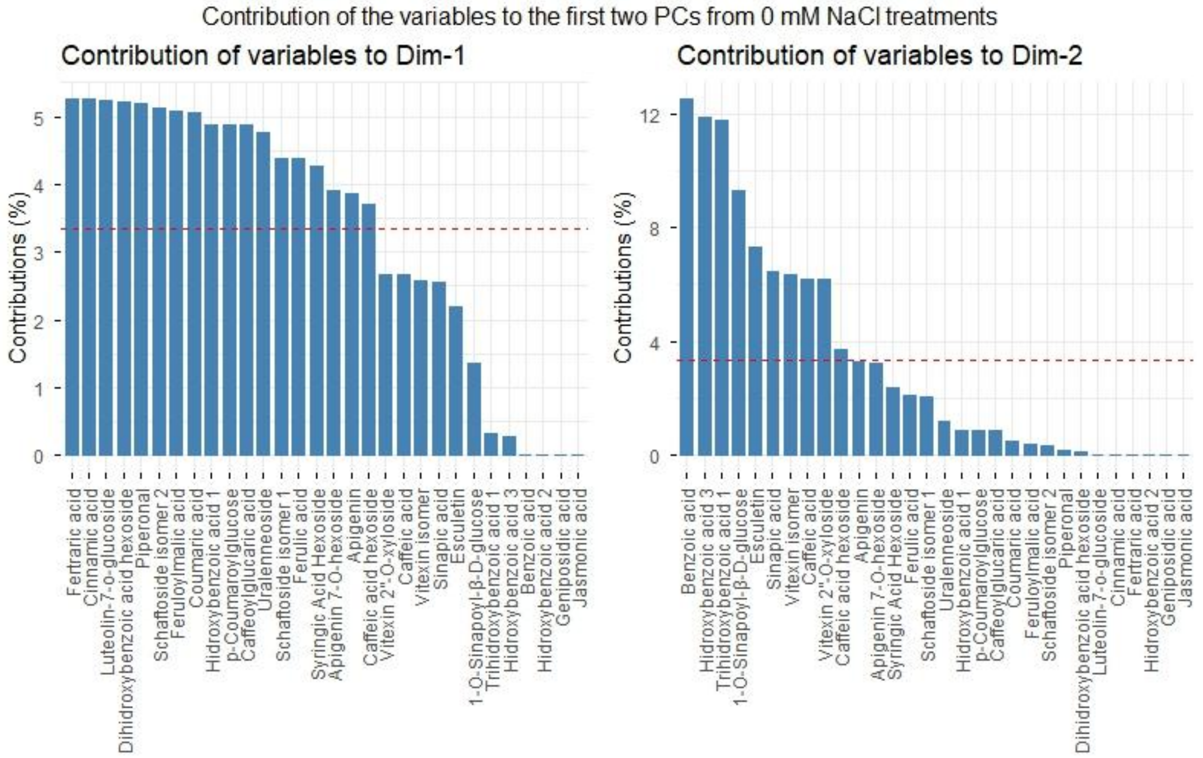

Figure S1. B: Contribution of the variables to the principal components for 0 mM NaCl treatment.

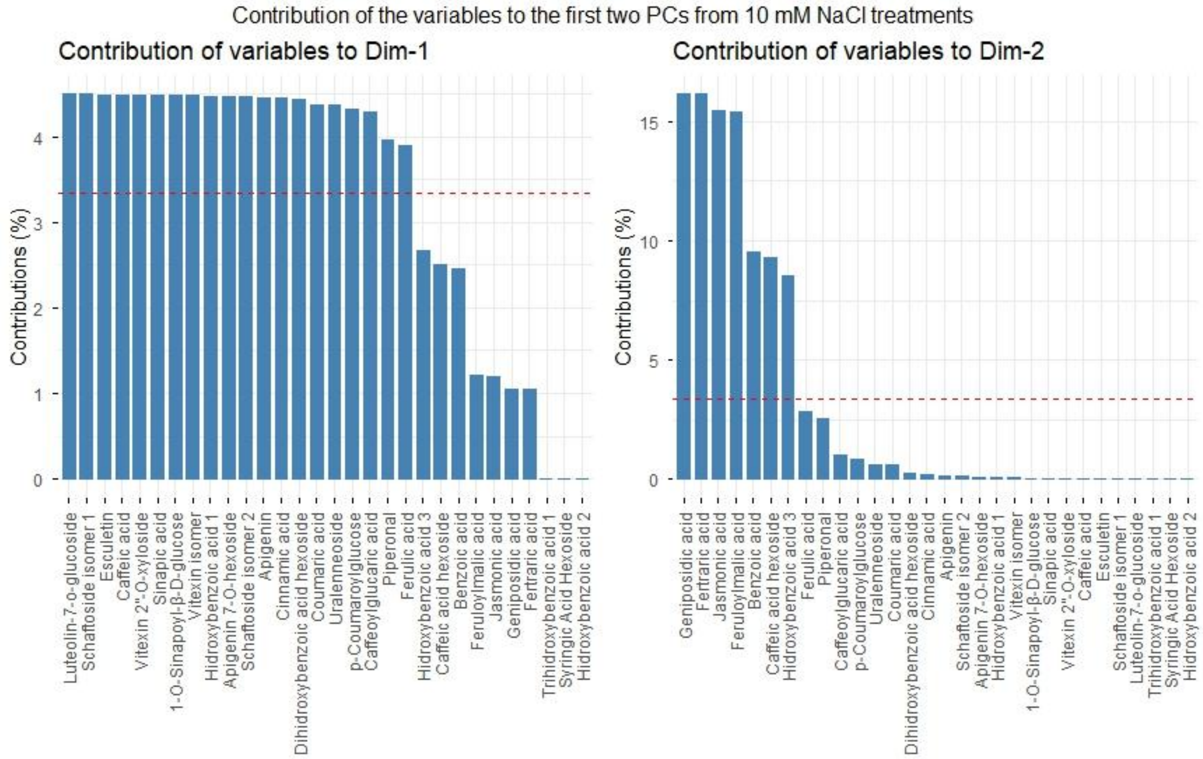

Figure S1. C: Contribution of the variables to the principal components for 10 mM NaCl treatment.

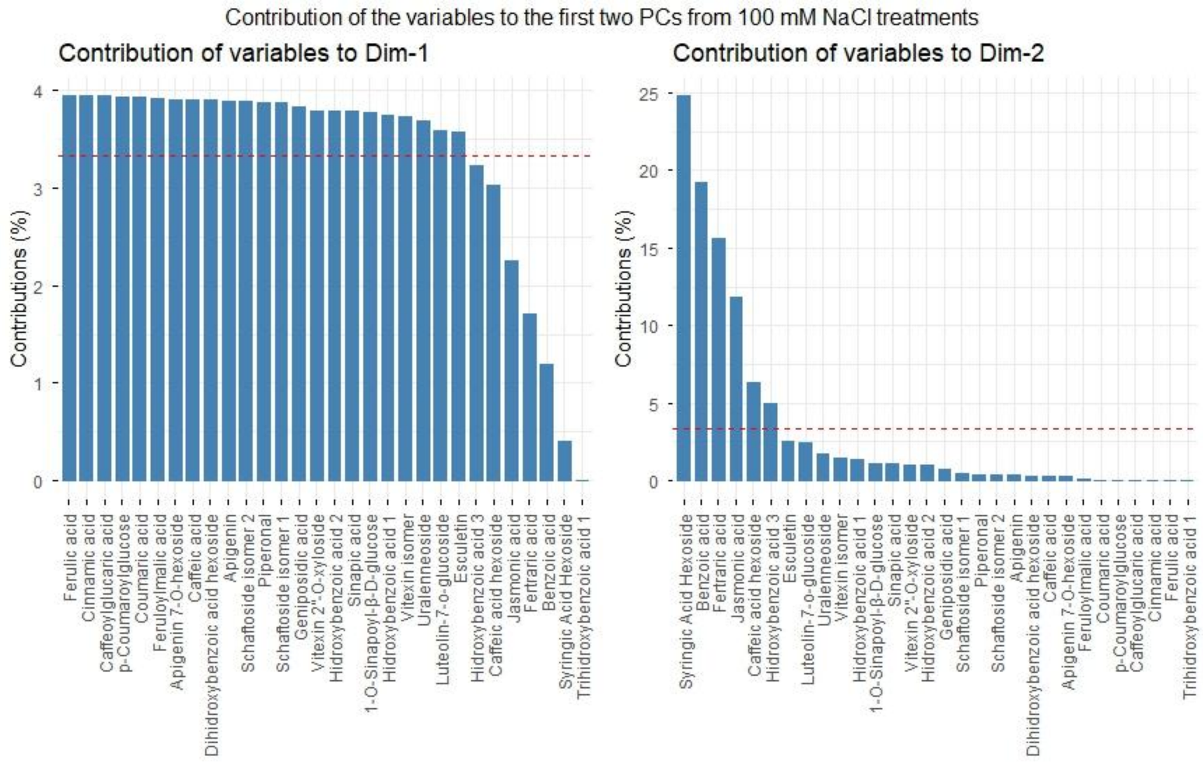

Figure S1. D: Contribution of the variables to the principal components for 100 mM NaCl treatment.

Supplementary Figure S2. Raman Spectroscopy

In the present samples, Raman spectra were acquired primarily as a complementary characterization technique. Multiple acquisition and processing approaches were explored in an effort to improve spectral quality; however, strong fluorescence consistently dominated the Raman response of all samples and characteristic ZnO phonon modes could not be resolved with sufficient confidence (Fig. 2.1). From the raw data, the multi-fold increase in fluorescence background was detected in 0<10<100 mM order (Fig. 2.2.).

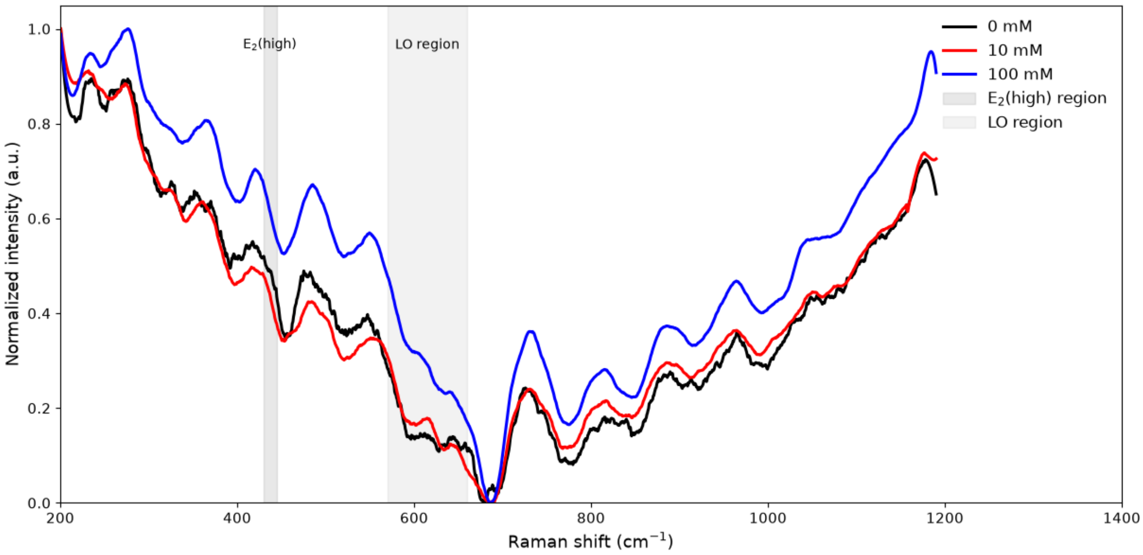

Figure S2. 1. Raman spectra of ZnO NPs biosynthesized using *Lemna minor* extracts obtained from plants cultivated under 0, 10, and 100 mM NaCl with expected positions of E_2 and LO region. Spectra were acquired using a high-resolution micro-Raman microscope equipped with a deep air-cooled EMCCD detector and a 785 nm excitation laser. Baseline correction and normalization were applied to facilitate comparison of spectral features among samples.

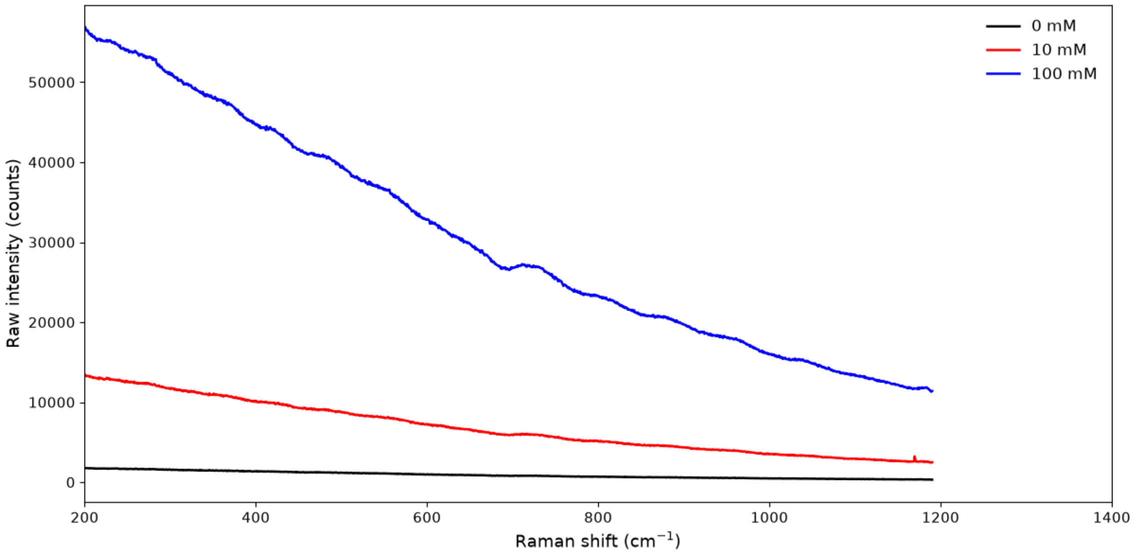

Figure S2. 2. Raw Raman spectra of three green ZnO nanomaterial samples.

Materials and methods

2. Raman Spectroscopy

Raman spectroscopic analysis of the synthesized samples was performed using a high-resolution Raman microscope equipped with 532 nm and 785 nm excitation lasers and Raman imaging capabilities. The instrument comprised a micro-Raman microscope with micrometer-scale spatial resolution and a deep air-cooled EMCCD detector. Spectra were acquired using selectable diffraction gratings (600, 1200, 1800, and 2400 g/mm), with adjustable spectrometer positions and cumulative signal acquisition. Confocal measurements were performed using selectable pinholes to improve spatial resolution and suppress background fluorescence. Raman imaging was available through predefined measurement grids, enabling spatially resolved spectral analysis of the samples. Raman spectra were baseline-corrected using the asymmetric least-squares method, smoothed using a Savitzky–Golay filter, normalized to the maximum intensity, and visualized in Python 3.14 using the NumPy, SciPy, Pandas, and Matplotlib libraries.
